# Alpha-boswellic acid accelerates acute wound healing via NF-κB signaling pathway

**DOI:** 10.1371/journal.pone.0308028

**Published:** 2024-09-03

**Authors:** Fang Dong, Lijuan Zheng, Xuanfen Zhang

**Affiliations:** 1 Department of Plastic Surgery, Lanzhou University Second Hospital, Lanzhou, China; 2 Digestive Department, Gansu Provincial Hospital, Lanzhou, China; Pennsylvania State University Hershey Medical Center, UNITED STATES OF AMERICA

## Abstract

**Background:**

Boswellic acids (BAs) showed promising effects in cancer treatment, immune response regulation, and anti-inflammatory therapy. We aimed to assess the roles of alpha-BA (α-BA) in treating acute wound healing.

**Methods:**

In vivo wound-healing models were established to evaluate the therapeutic effects of α-BA. Cell assays were conducted to assess the impact of α-BA on cellular biological functions. Western blot analysis was employed to validate the potential mechanisms of action of α-BA.

**Results:**

Animal models indicated that wound healing was notably accelerated in the α-BA group compared to the control group (P < 0.01). Hematoxylin and eosin (HE) staining and enzyme-linked immunosorbent assay (ELISA) assay preliminarily suggested that α-BA may accelerate wound healing by inhibiting excessive inflammatory reactions and increasing the protein levels of growth factors. Cell function experiments demonstrated that α-BA suppressed the proliferation and migration ability of human hypertrophic scar fibroblasts (HSFBs), thereby favoring wound healing. Additionally, α-BA exerted a significant impact on cell cycle progression. Mechanistically, the protein levels of key genes in nuclear factor kappa beta (NF-κB) signaling pathway, including cyclin D1, p65, IκBα, and p-IκBα, were downregulated by α-BA.

**Conclusions:**

α-BA demonstrated the ability to counteract the abnormal proliferation of skin scar tissues, consequently expediting wound healing. These findings suggest its potential for development as a new agent for treating acute wound healing.

## Introduction

Skin wound healing represents a multistep and intricate biological process, primarily categorized into four phases: hemostasis, inflammation, proliferation, and tissue remodeling [1]. Fibroblasts play a pivotal role in this process. Upon skin injury, fibroblasts swiftly aggregate at the wound site to facilitate thrombus formation. Consequently, they secrete fibrillar collagen and other components, accelerating extracellular matrix synthesis, repairing wound defects, and promoting wound healing [2]. During the inflammation phase, fibroblasts partake in phagocytosis, clearing cellular debris, necrotic tissues, and other inflammatory mediators [3]. Additionally, they actively secrete various growth factors and cytokines, stimulating angiogenesis and tissue regeneration [4, 5]. However, excessive fibrosis, infiltrating inflammatory cells, and abnormal extracellular matrix deposition can lead to abnormal skin scar tissue proliferation [6]. Therefore, medications capable of reversing this aberrant process hold significant promise for facilitating proper and expeditious wound healing.

Boswellic acid (BA) class of compounds, extracted from the gum resin of Boswellia species, principally comprises alpha-BA (α-BA), beta-BA (β-BA), 11-keto-β-BA (KBA), and 3-O-Acetyl-11-keto-β-BA (AKBA) [7]. These compounds have demonstrated promising effects in cancer treatment, immune response regulation, and anti-inflammatory therapy [8–12]. Owing to their potent biological activity, a majority of studies have concentrated on β-BA and its derivatives [13, 14]. In contrast, investigations concerning the therapeutic properties of α-BA have been relatively limited. Zhang et al. observed that α-BA played a protective role against ethanol-induced gastric ulcers through the Nrf2/HO-1 pathway [15]. Similarly, in Sprague-Dawley rat models, α-BA was found to expedite the healing of diabetic foot ulcers by suppressing inflammatory responses and enhancing angiogenesis [16]. However, the therapeutic efficacy of α-BA in acute wound healing has yet to be fully elucidated.

In this study, our aim was to explore the potential application of α-BA in acute wound healing treatment and conduct a preliminary investigation into its potential mechanisms.

## Material and method

### Wound-healing model

Male Sprague–Dawley rats at 8 weeks of age were utilized to establish the wound-healing model. Following anesthesia with sodium pentobarbital (50mg/Kg), hairs on the back of each rat were removed and the dorsal skin of each rat was sterilized with 75% ethanol thrice. A circular area of full-thickness skin (approximately 500 mm2) was excised to simulate the wound. Twenty-four rats were randomly divided into the control group (n = 12) and α-BA group (n = 12). The control group underwent daily applications of a 0.1% iodophor solution on the wound. α-BA was purchased from Nakeli Biotechnology (Chengdu, China; CAS Number: 471-66-9), dissolved in 10% dimethyl sulphoxide (DMSO) to create a storage solution, and subsequently diluted with normal saline to achieve a 3% working solution. The α-BA group received daily applications of a 3% α-BA working solution on the wound. Each group of rats was further randomly divided into three subgroups, which were sacrificed on days 3, 7, and 14, respectively. Wound healing progress was documented on days 3, 7, and 14 using a digital camera, and the healing rate was subsequently calculated. Additionally, body weights were recorded at day 0, 3, 6, 9, 12, and 14. All methods were carried out in accordance with relevant guidelines and regulations. All animal experiments were approved by The Ethical Committee of Lanzhou University Second Hospital. The data collection and analysis conformed to all local laws and were compliant with the principles of the Declaration of Helsinki.

### Hematoxylin and eosin (HE) staining

HE staining was conducted following standard protocols. After dewaxing and dehydration, rat wound tissues were stained using an HE staining kit (Solarbio, Beijing, China), and images were captured using an IX-90 confocal laser scanning microscope (Olympus Optical, Tokyo, Japan).

### Enzyme-linked immunosorbent assay (ELISA)

In the dose-exploratory phase, supernatants from HSFBs exposed to different α-BA concentrations were collected. ELISA assays were performed a rat TNFα ELISA kit (SER06411A; SABiosciences) and IL-6 ELISA kit (SER06481A; SABiosciences). In animal experiment, skin tissues (50mg) were added into 1ml lysates buffer to make homogenate. ELISA assays were performed using TGFβ1 ELISA kit (R&D System), FGF2 ELISA kit (Catalog no. ab100670, Abcam), and EGF ELISA kit (Catalog no. ab239424, Abcam), following the manufacturer’s instructions.

### Cell culture and grouping

Human hypertrophic scar fibroblasts (HSFBs) generously provided by Dr. Chuangang Tang were maintained in Dulbecco’s modified Eagle’s medium (DMEM) supplemented with 10% fetal bovine serum (FBS) and 1% penicillin/streptomycin under 5% CO2 at 37°C. In the dose-exploratory phase, α-BA was utilized at four concentrations: 100ug/ml, 200ug/ml, 300ug/ml, and 400ug/ml. For the formal experiment phase, an optimal concentration of 100ug/ml was chosen. Cell assays were categorized into three groups: blank control group, negative control group, and α-BA-treated group. The blank control group was cultured without any treatment, designated as “control” in subsequent analyses. The negative control group was cultured with a lipopolysaccharide (LPS) concentration to simulate inflammation, labeled as “LPS”. The α-BA-treated group was cultured with 0.5ug/ml LPS and 100ug/ml α-BA, and labeled as “LPS + α-BA”. The following cell experiments were performed at 48 hours after the LPS or“LPS + α-BA” administration.

### Transwell migration assay

HSFBs were cultured in a 96-well Transwell-plate with serum-free DMEM at a concentration of 5×103 per well. Complete medium containing 10% FBS was added to the lower chambers of the Transwell. After 24 hours of incubation, non-migrated cells were removed by a gentle swabbing process. Subsequently, migrated cell numbers were determined after fixation and staining, using a microscope.

### Cell Counting Kit-8 (CCK8)

Three groups of HSFBs with 5 replicate wells were cultured in a 96-well plate at a concentration of 5×103 per well. Following a 24-hour incubation period, 10 μl of CCK8 reagent (Dojindo, Shanghai, China) was added, and the absorbance was measured at 450nm.

### Real-time quantitative PCR (RT-qPCR)

The mRNA expression levels of cyclin D1 were determined by RT-qPCR according to a previously described method [17]. The primer sequences were as follows: cyclin D1 forward primer: GCTGCGAAGTGGAAACCATC; reverse primer: CCTCCTTCTGCACACATTTGAA; glyceraldehyde 3 phosphate dehydrogenase (GAPDH) forward primer: GGAGCGAGATCCCTCCAAAAT; reverse primer: GGCTGTTGTCATACTTCTCATGG.

### Cell cycle experiments

Cell cycle experiments were conducted using a cell cycle and apoptosis analysis kit (Beyotime, Shanghai, China) In brief, HSFBs was stained with a red fluorescent dye Propidium Iodide. DNA content of stained cells was analyzed utilizing a FACS Calibur flow cytometer (Becton Dickinson, NJ, USA).

### Western blot

Western blotting was performed as previously delineated [18]. The antibodies used included cyclin D1(1:1,000; Catalog no. ab16663; Abcam), p65 (1:1,000; Catalog no. ab16502; Abcam), IκBα (1:500; Catalog no. ab76429; Abcam), p-IκBα (1:10,000; Catalog no. ab133462; Abcam), GAPDH (1:5,000; Catalog no. ab8245; Abcam), and IgG secondary antibody (1:5,000; Catalog no. ab6789; Abcam).

### Statistical analysis

Statistical analysis involved the application of independent sample t-tests for two-sample comparison and chi-square tests for assessing healing rates. Graphs depicting statistical data were generated using GraphPad Prism 8 (GraphPad Software, Inc.), with significance levels indicated as follows: *P < 0.05; **P < 0.01; ***P < 0.001.

## Results

### α-BA accelerated wound healing in vivo

**Fig 1:**
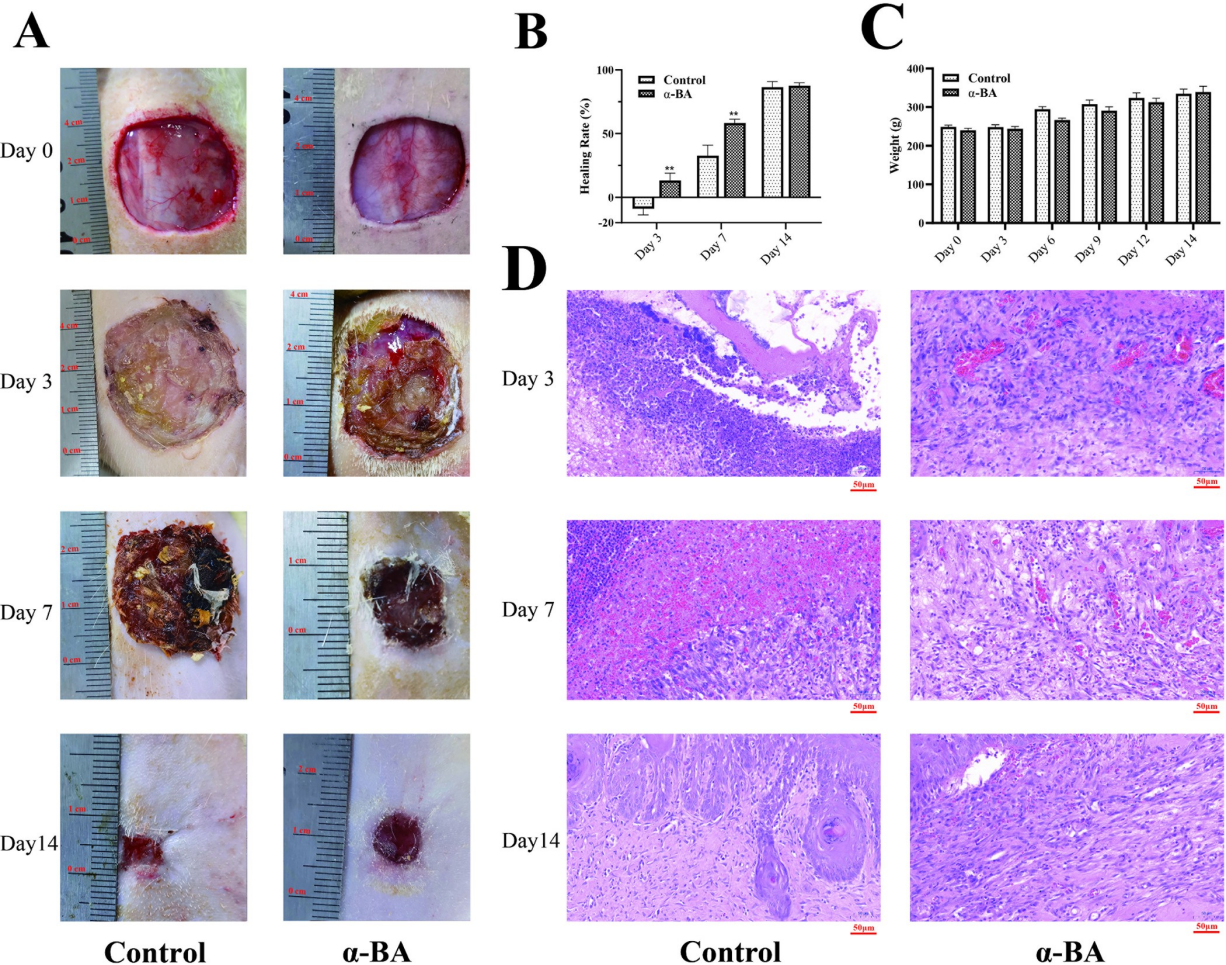
Wound-healing models. (A) Eight rats were randomly divided into the control group (n = 4) and α-BA group (n = 4). Wound healing progress was documented on days 3, 7, and 14 using a digital camera, and the healing rate was subsequently calculated. (B) Statistical graphs of changes in wound area during the healing process. (C) Body weight variations during the wound healing process. (D) Histological changes during the wound healing process (representative images). ** P < 0.01.

First, we established wound-healing models to investigate whether α-BA impacted the wound healing. As depicted in Fig 1A, the wound area in the control group initially increased and subsequently decreased until complete wound closure. Conversely, in the α-BA group, a consistent decrease in wound area was observed, with significantly higher healing rates on day 3 and 7 compared to the control group (Fig 1B, P < 0.01). Throughout the healing process, there were no significant differences in weight changes between the two groups (Fig 1C, P > 0.05), indicating that neither the wound nor α-BA treatment led to discernible weight loss in the rats.

Histological examination on day 3 revealed a substantial presence of inflammatory cells along with limited granulation tissue in the wounds (Fig 1D). In contrast, the α-BA group displayed abundant visible fresh granulation tissue and fewer inflammatory cells. By day 7 post-modeling, the control group exhibited extensive infiltration of inflammatory cells, increased proliferation of granulation tissue, and limited angiogenesis and fibroblast biogenesis. Conversely, the α-BA group presented conspicuous epithelial cells and granulation tissue, accompanied by well-organized fibroblasts and moderate inflammatory cell infiltration. By day 14, both groups primarily healed, with a small number of inflammatory cells still visible on the surface of the control group, while the α-BA group demonstrated nearly complete re-epithelialization and partial regeneration of skin appendage structure.

**Fig 2:**
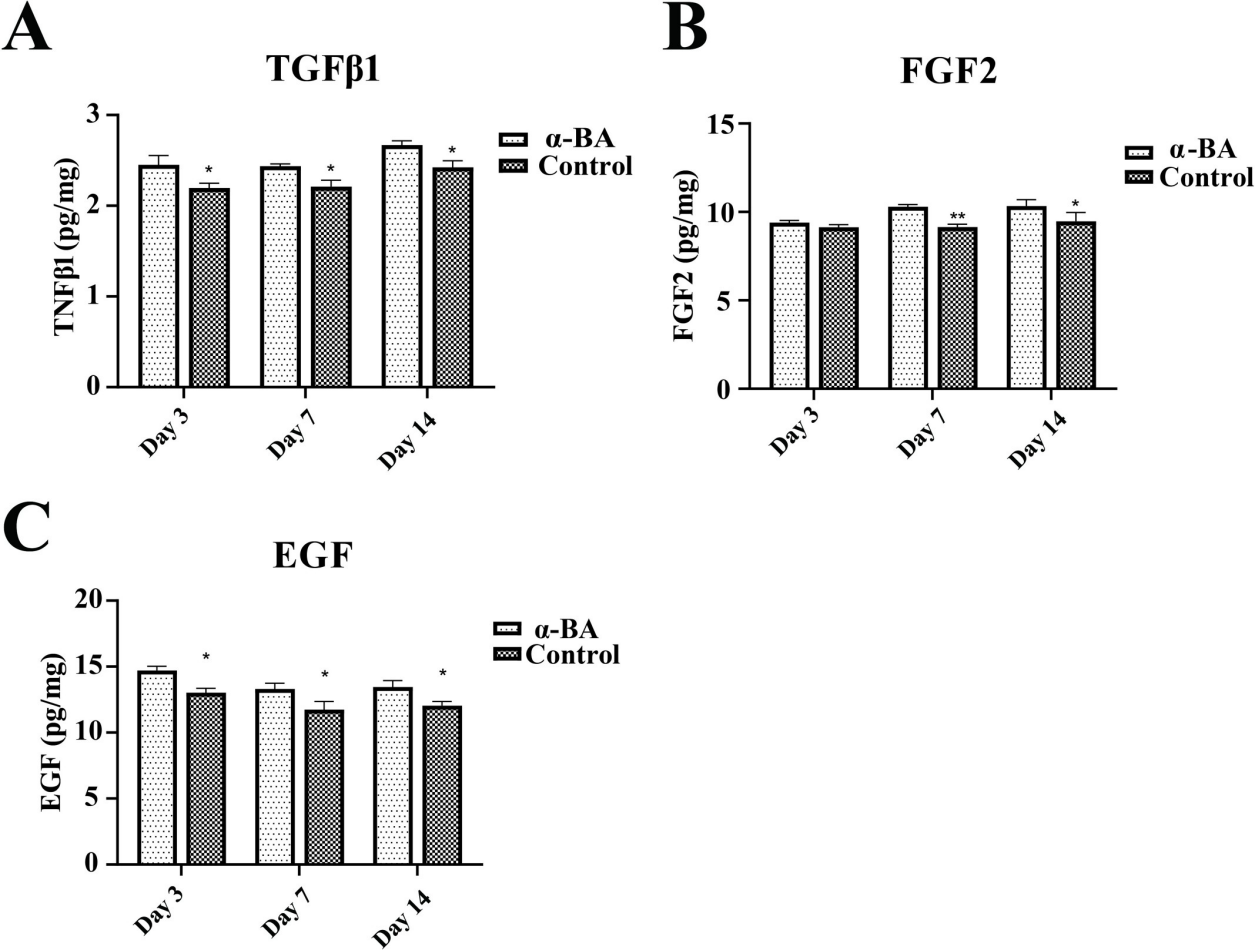
ELISA assays. (A) Changes in TGFβ1 protein level during the wound healing process. (B) Changes in FGF2 protein level during the wound healing process. (C) Changes in EGF protein level during the wound healing process. *P < 0.05. ** P < 0.01.

Furthermore, we examined the impact of α-BA on various growth factors on day 3, day 7, and day 14. The protein levels of TGFβ1, FGF2, and EGF were significantly elevated in the α-BA group compared to the control group, suggesting that α-BA facilitated fibroblast growth through augmented release of various growth factors, consequently expediting wound healing (Fig 2A–2C, P < 0.05).

### α-BA accelerated wound healing in vitro

**Fig 3:**
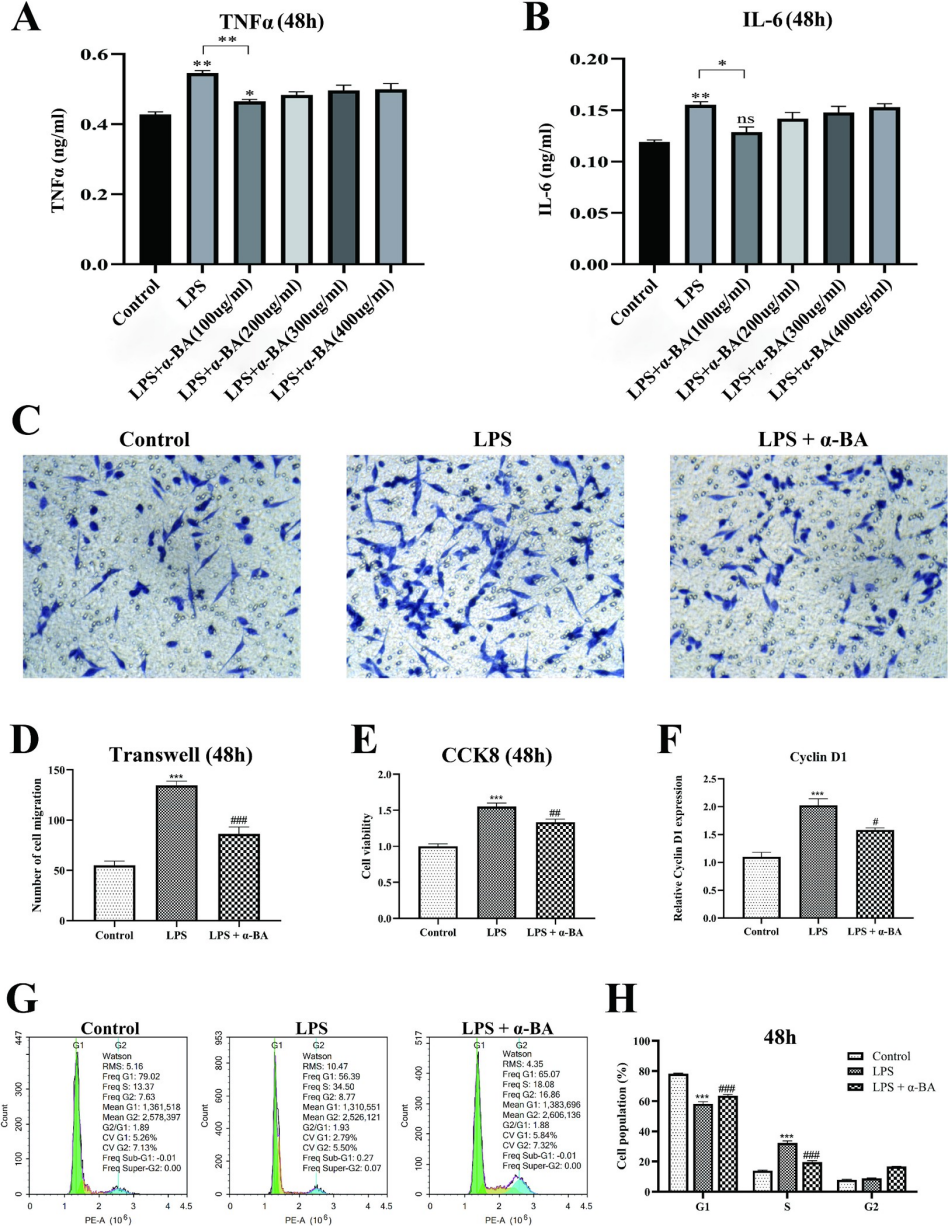
Determination of the optimal concentration of α-BA and cell experiments. (A) Changes in TNFα protein level during the wound healing process at different concentrations of α-BA. The optimal concentration of α-BA was 100ug/ml. (B) Changes in IL-6 protein level during the wound healing process at different concentrations of α-BA. (C) Transwell migration assay to assess the impact of α-BA on the cell invasion capacity (48h). (D) Statistical graphs of Transwell migration assay results. (E) CCK8 assays to assess the impact of α-BA on the proliferative activity (48h). (F) Changes in Cyclin D1 mRNA levels induced by α-BA. (G) Representative images of cell cycle experiments. (H) Statistical graphs of cell cycle experiments. *P < 0.05 (control vs. LPS). ** P < 0.01 (control vs. LPS). *** P < 0.001 (control vs. LPS). # P < 0.05 (control vs. LPS + α-BA). ## P < 0.01 (control vs. LPS + α-BA). ### P < 0.001 (control vs. LPS + α-BA). ns: not statistically significant.

The above animal model suggested that α-BA has already exhibited an anti-inflammatory effect within a maximum of 3 days. Generally speaking, the effect of drugs on individual cells is faster compared to animal models. Therefore, we examined the impact of varying concentrations of α-BA on inflammatory cytokines at 48h. The ELISA assay indicated that LPS significantly increased the production of inflammatory cytokines including TNFα and IL-6 48 hours after the LPS administration (Fig 3A and 3B). Notably, treatment with α-BA mitigated the inflammatory response induced by LPS, displaying optimal efficacy at a concentration of 100ug/ml.

Next, we investigated the impact of α-BA on the biological behavior of HSFBs. At 48 hours after the drug administration, Transwell migration assay demonstrated a substantial enhancement in the cell migration capability of HSFBs upon LPS treatment (Fig 3C and 3D, P < 0.001, vs. control), which was significantly reversed by α-BA (Fig 3C and 3D, P < 0.001, vs. LPS). Similarly, LPS enhanced the cell proliferation ability of HSFBs, while α-BA treatment notably decreased this trend, leading to significantly reduced cell viability (Fig 3E, P < 0.01, vs. LPS). Intriguingly, α-BA exhibited a significant inhibitory effect on cyclin D1, an essential cell cycle regulator, at the mRNA level (Fig 3F, P < 0.05). Subsequent cell cycle experiments revealed a higher proportion of S-phase cells and lower proportion of G1-phase cells in the LPS group compared to the control group, signifying distinctive proliferative and inflammatory characteristics (Fig 3G and 3H, P < 0.001). Conversely, the LPS + α-BA group exhibited an increased proportion of G1-phase cells relative to the LPS group, accompanied by a decreased proportion of S-phase cells. This collective evidence suggests that α-BA effectively suppressed the proliferation and migration abilities of HSFBs, thereby favoring wound healing.

**Fig 4:**
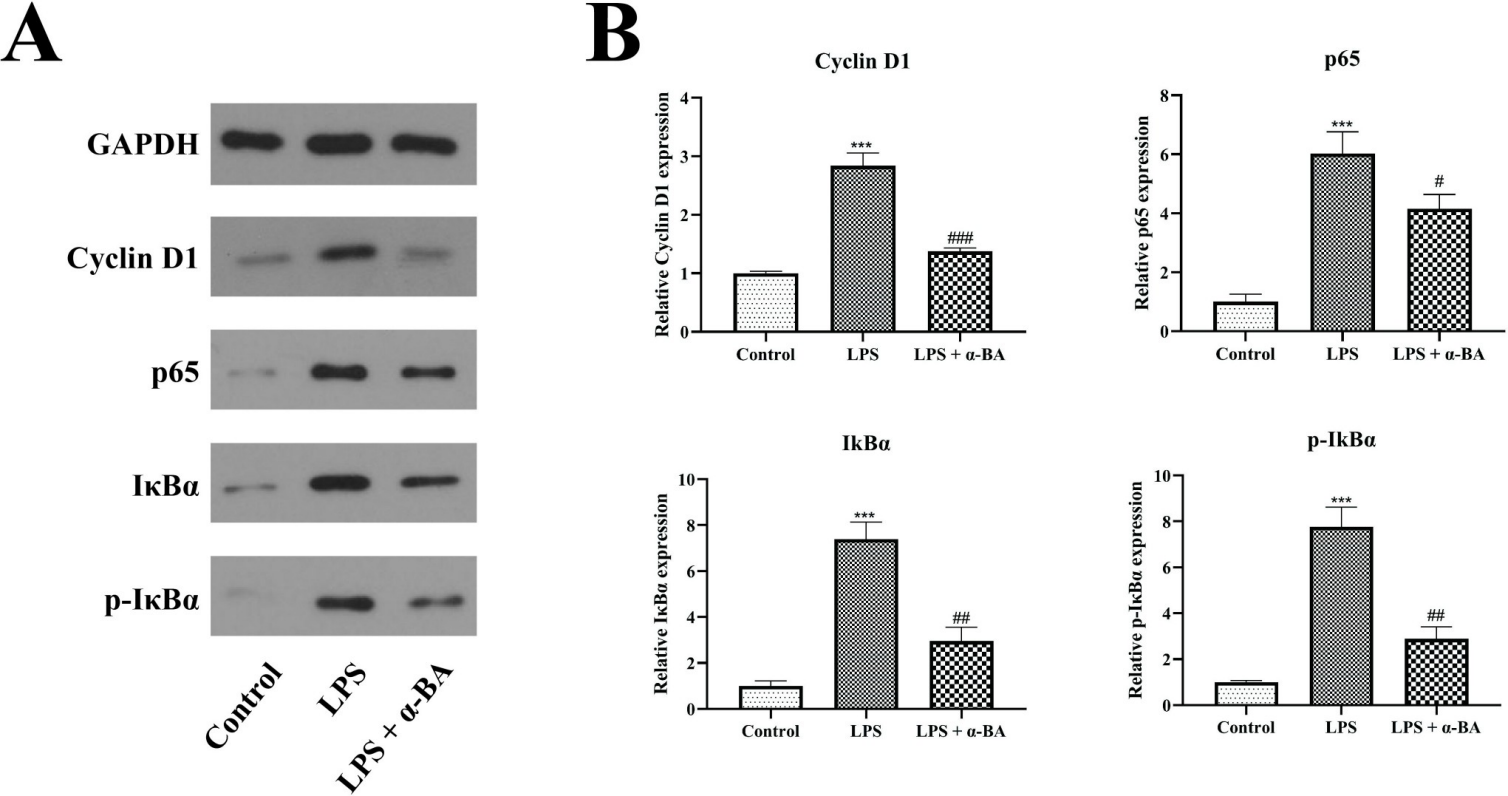
Mechanism of action of α-BA. (A) Western blot for NF-κB signaling pathway-related proteins. (B) Statistical graphs of Western blot. *P < 0.05 (control vs. LPS). ** P < 0.01 (control vs. LPS). *** P < 0.001 (control vs. LPS). # P < 0.05 (control vs. LPS + α-BA). ## P < 0.01 (control vs. LPS + α-BA). ### P < 0.001 (control vs. LPS + α-BA).

Given the involvement of the NF-κB signaling pathway, a canonical proinflammatory signaling cascade, in the wound healing process [19], and the promotion of cyclin D1 expression, we sought to investigate its potential modulation by α-BA. Western blot analysis indicated downregulation of key genes in the NF-κB signaling pathway, including cyclin D1, p65, IκBα, and p-IκBα, by α-BA relative to the LPS group (Fig 4A and 4B, P < 0.05).

## Discussion

The process of wound healing involves the participation of various cell types, with distinct cell populations involved at different phases. The appropriate number and activity of fibroblasts play a pivotal role in wound healing. However, uncontrolled proliferation and activation of fibroblasts can lead to abnormal scar formation. Consequently, the quest for affordable, effective pharmacological agents with minimal adverse effects has become an imperative concern. In our current investigation, we established rat models of acute wound healing to assess the impact of α-BA. Our results revealed that α-BA effectively inhibited the proliferation and migration abilities of HSFBs, concurrently reducing the number of cells in the S-phase, thereby reversing the uncontrolled proliferation and activation of fibroblasts, and, fostering a conducive environment for normal and rapid wound healing. To the best of our knowledge, this study represented the initial report on the therapeutic effects of α-BA in the treatment of acute wound healing. Our findings may inspire novel strategies aimed at mitigating scar formation and enhancing the quality of life for patients.

In comparison with other BAs, α-BA demonstrated relatively favorable pharmacological performance. Preliminary pharmacokinetic studies confirmed that α-BA became available eight hours after dosing, indicating high bioavailability [20]. Furthermore, the half-life of BAs was six hours, and steady-state levels were achieved approximately 30 hours post-treatment [21]. Additionally, the safety profile of BAs has been substantiated through numerous clinical trials involving osteoarthritis, irritable bowel syndrome, Crohn’s disease, and cerebral edema [22–26]. These advantageous pharmacological attributes and established safety profiles render α-BA a promising candidate for drug development.

Several signaling pathways have been implicated in wound healing. The Notch signaling pathway has showcased beneficial effects on endothelial, keratinocyte, and fibroblast cells during the wound healing process [27]. Activation of the Nrf2 signaling pathway has been associated with accelerated wound healing through the attenuation of cellular stress and augmentation of cellular antioxidant capacity [28]. Previous reports have also indicated the involvement of α-BA in wound healing via the Nrf2 pathway [15]. However, alterations in cellular factors and protein levels related to wound healing suggested a significant influence of α-BA on cyclin D1, a critical NF-κB target gene. Multiple studies have underscored the crucial involvement of the NF-κB signaling pathway in the wound healing process [29, 30]. Thereforee, it was identified as a potential mechanism underlying the effect of α-BA in our investigation. Indeed, the activity of the NF-κB pathway was significantly modulated by α-BA. Although our study confirmed the impact of α-BA through the NF-κB pathway, it is conceivable that other signaling pathways may also be implicated. Future studies employing whole-genome sequencing and pathway enrichment analysis hold promise in elucidating the predominant mechanisms underlying the role of α-BA in wound healing.

However, our study had some limitations. Firstly, only animal models were utilized in our analysis. Previous clinical trials have predominantly focused on other types of BAs and other inflammatory diseases. Rigorous clinical trials focused on α-BA in acute wound healing would be essential to validate its safety and efficacy. Secondly, our study specifically investigated fibroblasts, numerous other cell types, including neutrophils, macrophages, and endothelial cells, may also be impacted by α-BA. Thirdly, our efforts were limited to the detection of a few common cytokines and proteins. A comprehensive proteomic analysis would offer more robust evidence for comprehending the pathological and physiological changes and mechanisms underlying the wound healing process.

## Supporting information

S1 Data (ZIP)

S1 Raw images (PDF)
