# Supplementary figures and images for: Alpha-boswellic acid accelerates acute wound healing via NF-κB signaling pathway
Source: PLoS One. 2024 Sep 3;19(9):e0308028. doi: 10.1371/journal.pone.0308028 (PMC11371135; doi:10.1371/journal.pone.0308028)

Figure 4A GAPDH

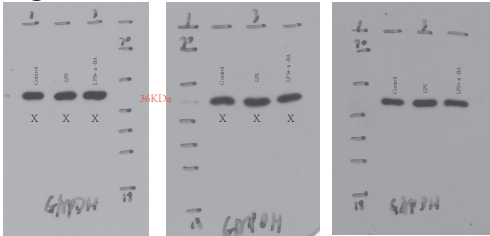

Figure 4A Cyclin D1

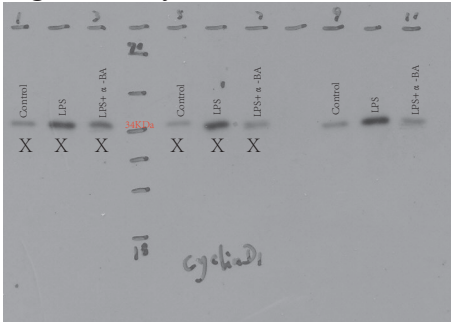

Figure 4A p65

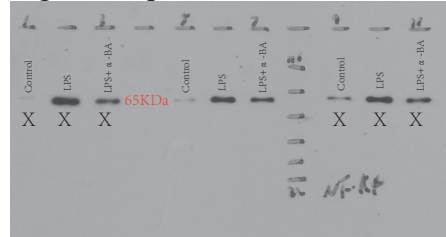

Figure 4A IκBα

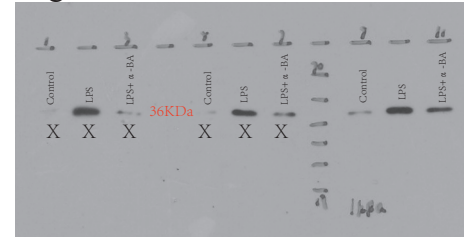

Figure 4A p-IκBα

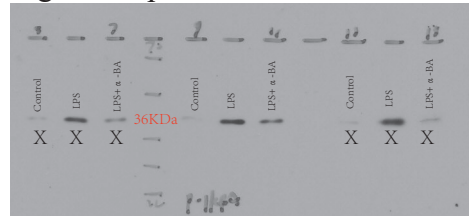

Supplement: S1 Raw images — (PDF) [file pone.0308028.s002.pdf]
